# Antiseptic Treatment of Wounds

**Published:** 1870-08

**Authors:** 


### Editorial Department.

### Antiseptic Treatment of Wounds.

Numerous inquiries, as to the possibility of obtaining direct union in lacerated wounds, and if so, of the best methods of procedure to obtain such results, lead to a very brief review of this subject, and reply to these important surgical questions. The foreign Medical Journals have been filled with reports of cases treated "antiseptically," with especially favorable results; and the American Periodicals also contain similar views, or reprints of the same. Joseph Lister, of the Edinburgh Royal Infirmary, in a recent lecture, pubished in the *Lancet*, relates cases of compound dislocation of the ankle, and compound fracture, treated antiseptically, with very favorable results; but these results, favorable as they are, are not better, or in any respect unlike those often observed where no such parade of treatment is adopted. It may be that his plan, modified to suit the views of different practitioners, has its advantages, and, with a view of placing it before our readers, we make as condensed an abstract as consistent with an understanding of his views and plans of procedure.

Speaking of a case of compound fracture of the tibia, and dislocation of the ankle joint, he says: "For the purpose merely of facilitating the return of the protruding end of the fibula, I nipped off a portion of it with cutting pliers, and, with the same object, enlarged slightly with scissors the lower end of the rent in the skin, which opposed a barrier to its passage. But to all intents and purposes the dislocation was simply reduced. The case, however, was treated antiseptically. Watery solution of carbolic acid, as strong as it can be made (one part of the crystals to twenty of water), was thrown into the joint with a syringe, the edges of the skin being held together to prevent its escape, and cause its penetration to all the internal recesses of the wound; and this was further promoted by free manipulation of the injured part while the fluid was still in the interior. There was a time when we should have thought that to introduce an irritating liquid like this, into the ankle-joint, would be to take an unwarrantable liberty with the articulation. But we now understand that the transient irritation caused by the antiseptic lotion is nothing compared with the abiding influence of the far more acrid products of putrefaction. But when the injury has been received some time before you see the patient, and inflicted, as in the present instance, in a rude way, involving the chance of foreign material having been introduced and mixed, perhaps, with clots of blood lying in inaccessible recesses of the wound, it seems wise to employ as strong a solution as water will produce. The liquid introduced having been squeezed out, the process of injection and manipulation was performed a second time for greater security, and the skin in the vicinity having been previously well washed with the lotion, to destroy organisms adhering to it or the hairs, an external dressing was applied. Lac plaster was wrapped in two layers round the limb, from three or four inches above the upper extremity of the wound to as far below the lower end—that is to say, extending well up the leg, and embracing the heel and instep, the foot meanwhile being held in good position. A cloth to absorb the blood and serum which would be discharged from beneath the margins of the plaster, was then bandaged on, and a splint applied to the inner aspect of the leg and foot. A well overlapping cap of lac plaster, in double layer, was then applied surrounded by a cloth, to absorb discharge, secured by bandage and pins. I cannot too strongly impress upon you the importance of having the plaster extend freely beyond the wound at every part, so that the discharge may have to travel a considerable distance beneath the impermeable antiseptic layer before reaching the sources of mischief externally. It is only in this way that you can guard securely against the spread of the putrefactive fermentation into the wound. Yet there is nothing in the antiseptic treatment that I find more apt to be neglected."

This may be, and we have no doubt is, a good method of treatment in the injury described, but the advantage seems to be greatly over estimated, not only by Mr. LISTER, but by others who have recently become so greatly enamored with carbolic acid, which, like nearly all other therapeutical agents, stimulates the highest expectations upon its first introduction to the notice of the profession. As a deodorant and disinfectant it certainly seems to have marked virtues, and in weak, watery solution makes, perhaps, one of the very best lotions for moistening the dressings in all wounds with lacerated incisions or broken surfaces. The question to be determined is,—will carbolic acid, injected into lacerated wounds, like those produced by compound dislocations and fractures, prevent "putrefaction," as Mr. LISTER calls it? and convert them into simple granulating surfaces. While we are very favorably impressed with the virtues of carbolic acid, and believe it is capable of doing much as an antiseptic, still we are not ready to conclude that lacerated wounds, even of the gravest character, depend upon its application for the very most favorable results. Who has not seen compound fracture heal as kindly and as early as even the most favorable cases of simple fracture? Who that has treated compound dislocation of the ankle joint with no attempts as disinfection, but has seen the most rapid recoveries? It is not uncommon to see all the injuries narrated as proving the sovereign power of carbolic acid, terminate as favorably under simple dressings as they are reported to terminate under the best directed antiseptic plan of procedure. Mr. SYME said, that on looking into the Hospital record, the last fourteen cases of compound fracture into the ankle joint, all terminated fatally. He, therefore, regarded amputation at the ankle as the best treatment in most cases; he, however, modified his treatment in some cases to sawing off the end of the tibia, making excision of the ankle. Such practice prevailed at one time, and amputation was made much more unhesitatingly than now. Ample experience has again and again demonstrated the propriety of returning compound dislocations and compound fractures, in almost all cases, and in the great majority of instances the results will be favorable. This was demonstrated before carbolic acid came into use, and would remain true, if carbolic acid should become unknown, so that the antiseptic treatment of lacerated wounds, and the protection it affords, cannot be placed as a reason for the safety and propriety of such practice.

In saying this, we are not to be understood as opposing the use of antiseptics in the treatment of lacerated wounds, or of carbolic acid, as a valuable agent for that purpose. We desire to place our therapeutics upon the basis of exact truth, unwilling to allow ourselves to be mistaken as to the true sources of our safety and strength in the treatment of this formidable and dangerous class of injuries.

